# Molecular identification of toxin-antitoxin system genes and their relationship with biofilm formation among clinical isolates of Escherichia coli obtained from hospitalized and outpatients in educational hospitals in Ahvaz, Iran

**DOI:** 10.3205/dgkh000665

**Published:** 2026-07-17

**Authors:** Arshid Yousefi Avarvand, Morteza Saki, Melika Moradi, Alireza Ekrami, Sousan Akrami

**Affiliations:** 1 Infectious and Tropical Diseases Research Center, Health Research Institute, Ahvaz Jundishapur University of Medical Sciences, Ahvaz, Iran; 2 Department of Laboratory Sciences, School of Allied Medical Sciences, Ahvaz Jundishapur University of Medical Sciences, Ahvaz, Iran; 3 Department of Microbiology, Faculty of Medicine, Ahvaz Jundishapur University of Medical Sciences, Ahvaz, Iran; 4 Department of Microbiology, School of Medicine, Tehran University of Medical Sciences, Tehran, Iran; 5 Students' Scientific Research Center (SSRC), Tehran University of Medical Sciences, Tehran, Iran

**Keywords:** toxin-antitoxin systems, biofilm, Escherichia coli, hospitals

## Abstract

**Background:**

Toxin-antitoxin (TA) systems are present on the chromosomes and plasmids of many bacteria, including *Escherichia coli*. The functions of TA systems in bacteria are unclear. The biological roles of TA systems are hypothesized to include growth regulation, persistence, and biofilm development. *E. coli* biofilms are the source of both urinary tract infections and bacteremia.

**Objectives:**

The current investigation aims to discover the relationship between biofilm development and toxin-antitoxin systems in clinical isolates of *E. coli*.

**Materials and methods:**

A total of 100 *E. coli* isolates were tested for biofilm formation by microtiter plate assay and the presence of several TA systems such as MazF, RelE, hipA, ccdB, and MqsR.

**Results:**

Microtiter plates revealed that 90 *E. coli* isolates produced biofilms. The results revealed that 75 (75%), 80 (80%), 81 (81%), 58 (58%), and 51 (51%) of the isolates contained mazF, ccdB, relE, mqsR, and hipA TA loci, respectively.

**Conclusions:**

The findings suggested that TA genes are common in clinical isolates of *E. coli* strains. The results demonstrated that the TA system is related with biofilm development.

## Zusammenfassung

**Hintergrund:**

Toxin-Antitoxin (TA)-Systeme befinden sich auf den Chromosomen und Plasmiden vieler Bakterien einschließlich *Escherichia (E.) coli*. Die Funktionen der TA-Systeme in Bakterien sind unklar. Es wird angenommen, dass die biologische Rolle der TA-Systeme die Wachstumsregulierung, die Persistenz und die Biofilmentwicklung umfasst. Biofilm bildende *E. coli* sind die Ursache von Harnweginfektionen und Bakteriämie.

**Zielsetzung:**

Untersuchung zielt darauf ab, den Zusammenhang zwischen Biofilmentwicklung und Toxin-Antitoxin-Systemen in klinischen Isolaten von *E. coli* zu untersuchen.

**Methode:**

100 *E. coli*-Isolate wurden mittels Mikrotiterplatten-Assay auf Biofilmbildung und das Vorhandensein verschiedener TA-Systeme wie MazF, RelE, hipA, ccdB und MqsR untersucht.

**Ergebnisse:**

90 *E.coli*-Isolate bildeten Biofilme. Die Ergebnisse zeigten, dass 75 (75%), 80 (80%), 81 (81%), 58 (58%) und 51 (51%) der Isolate die TA-Loci mazF, ccdB, relE, mqsR bzw. hipA enthielten.

**Schlussfolgerungen:**

Die Ergebnisse legen nahe, dass TA-Gene in klinischen Isolaten von *E. coli*-Stämmen weit verbreitet sind. Die Ergebnisse zeigten, dass das TA-System mit der Biofilmentwicklung zusammenhängt.

## Introduction

*Escherichia (E.) coli* [1] can cause infections in different parts of the body, such as the urinary tract, gastrointestinal tract, and bloodstream. These are significant health concerns, especially for hospitalized and immunocompromised patients. One major reason for *E. coli's* capability to thrive in different environments, including healthcare settings, is due to its ability to form biofilms. Biofilm formation enables the bacteria to attach to surfaces and resist antibiotics and the immune system, contributing to its prevalence in healthcare settings [2], [3]. 

To survive under unfavorable conditions, *E. coli* can adapt through the formation of biofilms, which is a complex process that involves numerous mechanisms, including the use of toxin-antitoxin (TA) systems. TA systems are present in many bacteria and are known to play a critical role in various bacterial behaviors, including programmed cell death, plasmid maintenance, and biofilm formation [4]. 

Toxin-antitoxin (TA) systems play a crucial role in biofilm formation in many bacterial species, including *E. coli*. These systems comprise two genes encoding a stable toxin and an unstable antitoxin. The antitoxin binds to the toxin, preventing its activity and ensuring cell survival. Under specific stress conditions, such as nutrient limitation or antibiotic exposure, the antitoxin becomes unstable and degraded, releasing the toxin to exert its effect and leading to cell death or latency [4], [5], [6]. TA systems are classified into six types based on their antitoxin molecular nature and mode of action. The most studied type-II TA systems consist of two classes: RNA antitoxins and protein antitoxins. RNA antitoxins, also known as small non-coding RNAs (sRNAs), inhibit toxin mRNA translation by base-pairing, while protein antitoxins bind directly to the toxin protein and neutralize its activity [7], [8], [9]. 

*E. coli* has several characterized TA systems, including mazEF, relBE, hipBA, ccdAB, and mqsRA, which play essential roles in biofilm formation, stress response, and antibiotic resistance. The molecular mechanism of action differs depending on the toxin, with some toxins, such as MazF, ChpBK, and YoeB, directly cleaving RNAs, while others, like RelE, cleave only translated mRNAs. Activation of mazEF and relBE results in different effects on bacterial physiology. The mazEF system promotes biofilm formation under various stress conditions by increasing the production of extracellular polymeric substances (EPS) and enhancing cell surface hydrophobicity. The relBE system is implicated in persistence, a dormant state that allows bacteria to survive exposure to antibiotics. Finally, the ccdAB system is involved in plasmid maintenance and contributes to the formation of antibiotic-resistant persister cells [10], [11]. 

In this study, we aimed to identify TA system genes (mazEF, relBE, hipBA, ccdAB, and mqsRA) and investigate their relationship with biofilm formation in clinical *E. coli* isolates obtained from hospitalized and outpatient settings in Ahvaz. The findings could contribute to the development of new strategies to combat *E. coli* infections by shedding light on the mechanisms underlying biofilm formation in *E. coli*.

## Material and methods

### Collection of bacterial isolates

100 non-duplicate clinical *E. coli* isolates were collected from teaching hospitals in Ahvaz in October 2021 and March 2023. The isolates were identified using conventional biochemical tests and stored at -80°C in tryptic soy broth (TSB) containing 20% glycerol [12]. The isolates were identified through polymerase chain reaction (PCR) by 16s ribosomal ribonucleic acid (rRNA) primers (16S-F, 5'-TGT GGG AAC GGC GAG TCG GAA TAC-3'; 16S-R, 5'-GGG CGC AGG GGA TGA AAC TCA AC-3') [13].

### Microtiter plate biofilm assay and Estimation of bacterial biofilm

A microtiter plate biofilm assay was conducted to evaluate the biofilm formation of the isolates. The isolates were incubated in (Luria-Bertani) LB broth overnight and adjusted to an optical density (OD) between 0.4-0.6 at 600 nm. Then, 190 µl of LB broth medium was added to each well of polyvinylchloride 96-well microtiter plates, and 10 µl of the bacterial suspension was added to each well. The isolates were continuously incubated overnight with shaking at 30 rpm at 37°C, and the biofilm assay was performed in triplicate for each isolate with LB broth medium as negative control. After incubation, the microplates were washed with distilled water, stained with 0.1% crystal violet, and then washed with distilled water for three times. The OD at 550 nm was measured with an enzyme-linked immunoassay (ELISA) reader, and averages and standard deviations were calculated for all experiments. For quantitative analysis of the biofilm production, the average absorbance from the control wells [10] was subtracted from the A550 nm of all test wells. Averages and standard deviations were calculated for all experiments. *Pseudomonas aeruginosa* PAO1 strain was used as a positive control for biofilm assay. The isolates were classified into four categories based on the average absorbance from the control wells: A≤Ac=no biofilm producer, Ac<A≤(2×Ac)=weak biofilm producer, (2×Ac)<A≤(4×Ac)=moderate biofilm producer and (4×Ac)<A=strong biofilm producer [12]. 

### Polymerase chain reaction amplification

PCR amplification was performed to screen all 100 isolates for the presence of the 5 TA-system genes, hipBA, ccdAB, mqsRA, mazEF,and relBE (Table 1 (Tab. 1)) [14], using a C1000TM Thermal Cycler (BIO RAD, USA). One colony from each isolate was cultured overnight in LB broth medium, and genomic DNA was subsequently extracted using the boiling method. PCR was subsequently performed in a total volume of 25 µl containing 1 µl PCR buffer, 2 mM MgCl2, 2 mM dNTPs, 10 pmol of primers, 0.25 U Taq DNA polymerase (CinnaGen Co, Iran) and 5 µl of template DNA. Then the PCR products were analyzed by electrophoresis on 1% (w/v) agarose gel containing DNA safe stain. Agarose gels were visualized, and OD at 280 nm was measured by gel documentation (Gel Doc™ XR+, USA) [12]. 

### Statistical analysis

Statistical analysis techniques were employed to examine the relationship between biofilm formation and TA genes. The data was represented using percentages, means, and standard deviations. The study used the Chi-squared and Fisher’s exact test to determine correlation, and the Monte Carlo method with 10,000 tables and a starting seed of 200,000 was used when the Chi-squared test was not applicable. Logistic regression was used to make adjustments, and statistical significance was set at P<0.05.

## Results

### Demographic information of patients

This cross-sectional study gathered and examined 100 *E. coli*-positive culture samples from patients. Among these 100 strains, 63 (63%) were obtained from male patients and 37 (37%) from female patients. The patients’ ages varied from 16 to 81 years, with 20% being between the ages of 16 and 20. In terms of hospital wards, the men's ward had the highest frequency of isolates (28%, 28/100), followed by the women’s ward (23, 23/100) and internal medicine (10, 10/100).

### Biofilm phenotypes

90 strains (90%) were capable of forming biofilms. Based on OD, the ability to form biofilms was classified into four categories: No biofilm formation, strong biofilm, moderate biofilm, and weak biofilm. Of the 100 strains tested, 10 (10%) were negative biofilm formers, 39 (40%) were weak biofilm formers, 36 (36%) were moderate biofilm formers, and 15 were strong biofilm formers.

### Prevalence of TA genes

Chromosomal DNA of all *E. coli* clinical isolates was subjected to PCR. The results were positive if PCR products of the expected size were observed on the agarose gel. The results showed that, of the 100 *E. coli* strains, 75 (75%), 80 (80%), 81(81%), 58(58%) and 51(51%) were positive for mazF, ccdB, relE, mqsR and hipA genes, respectively. Therefore, among the genes of the TA system, the frequency of the relE gene was the highest and the frequency of the hipA gene was the lowest. 

### Association between TA systems and biofilm formation

**Figure 1:**
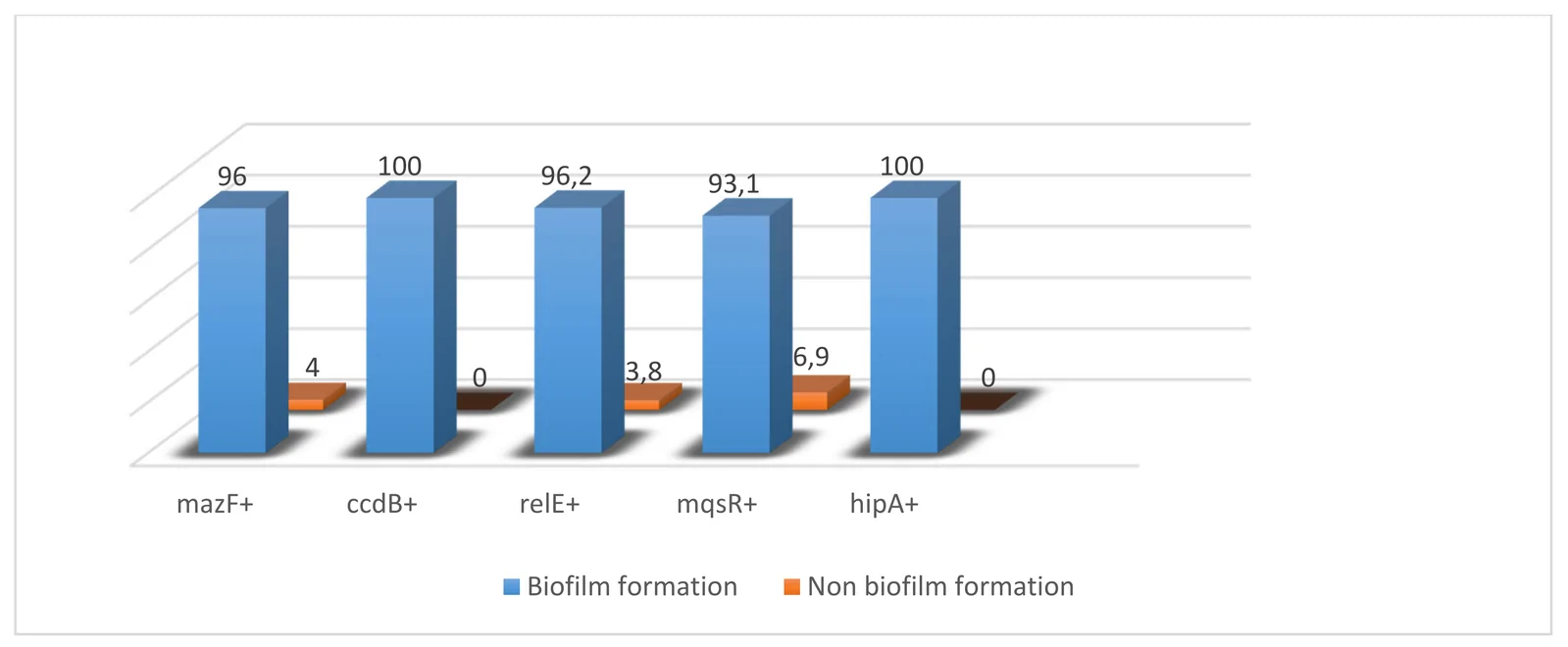
Frequency (%) of coexistence of toxin-antitoxin system genes and biofilm formation among 100 *E. coli* strains isolated

Among* mazF-*, *relE-*, *ccdB-*, *hipA-*, and *mqsR*-positive isolates, 96%, 96.2%, 100%, 100%, and 93.1% of *E. coli* clinical isolates were positive for biofilm formationby microtiter plate assay, respectively. These results showed a significant relationship between TA-positive isolates and biofilm formation (P<0.05), since biofilm formation among TA-positive isolates is more frequent than those of TA-negative isolates. The chi-squared statistic was used to test for the correlation between biofilm formation and TA systems (Figure 1 (Fig. 1)).

## Discussion

Little research has been performed in Iran on TA systems in bacteria. The purpose of this work was to identify TA system genes (mazEF, relBE, hipBA, ccdAB, and mqsRA) and evaluate their association with biofilm formation in clinical *E. coli* isolates collected from Ahvaz hospitals and outpatient settings. The study’s primary results include:

1. 90% of isolates may form biofilms;
2. a high frequency of toxin-antitoxin system genes such as mazF, ccdB, and relE; and
3. a substantial link between TA-positive isolates and biofilm formation.

Early studies revealed that TA systems did not play a role in biofilm development. For example, Lemos et al. [15] found that mutants of *Streptococcus mutans* that lacked homologues of the mazF and relE genes had no effect on biofilm formation when compared to parental strains. The first TA system found to be associated with biofilm development was mqsRA in *E. coli* [16]. The role of this TA system in biofilm development has been connected to mqsRA in motility, biofilm formation, and the autoinducer-2 quorum sensing system [17]. Kasari et al. [18] found a link between the mqsRA gene and biofilm development. Deleting the mqsRA gene significantly reduced biofilm formation. The mqsR gene is the most significantly elevated in *E. coli* persister cells, and it is the first TA system linked to biofilms. The deletion of mqsR gave the first indication that a single toxin could be eliminated and the number of persister cells decreased, thus tying toxins to persistence [19]. The MqsR tridimensional structure consists of an alpha/beta fold that is homologous with the RelE/YoeB toxins, while MqsA is an elongated dimer that neutralizes MqsR toxicity [20]. In addition to its activity as a typical antitoxin, MqsA acts as a global regulator. It is the first antitoxin demonstrated to regulate more than one locus by binding palindromic sequences at different chromosomal sites. MqsA regulates essential physiological processes, such as biofilm development and the overall stress response [21]. Interestingly, the MqsR/MqsA TAc system also controls the production of another toxin/antitoxin system, GhoT/GhoS, since MqsR preferentially cleaves the mRNA of antitoxin GhoS; hence, there is a hierarchy in TA systems that govern cell physiology [22]. The GhoT toxin, in turn, causes cell membrane damage, which reduces the creation of ATP-halting metabolism, protecting cells during stressful circumstances. MqsR/MqsA may play a physiological role in increasing bacterial survival in response to bile acid stress in the gastrointestinal tract, as MqsR degrades ygiS mRNA, which encodes a periplasmic protein that promotes the uptake of one of the main components of bile, i.e., deoxycholate salt. Degradation of ygiS mRNA will therefore lower the YgiS protein levels, reducing the absorption of deoxycholate and increasing tolerance to exposure [23]. TA systems are now divided into five types (I–V) based on the nature of the antitoxin and the method of interaction between the toxin and antitoxin. Type II mazF-mazE and rnlA-rnlB are implicated in phage inhibition because they strongly impede infection with phage P1 and phage T4, respectively [24].

In this study, the prevalence of five distinct TA-system genes was observed: mazF, ccdB, relE, mqsR, and hipA. The most common TA-system genes were relE (81%), ccdB (80%), and mazF (75%). Research from 2023 evaluated the incidence and genetic diversity of TA-system genes in *E. coli* isolates from patients in Iran. Those results showed that of the 67 *E. coli* strains, 48 (71.6%), 47 (70%), 52 (77.6%), 24 (35.8%) and 21 (31.3%) were positive for mazE, ccdAB, relB, mqsR and hipA genes, respectively [25]. However, the frequencies of TA-gene systems in our study exceeded those found by Roshani et al [25]. Research at a Chinese hospital explored the role of ccdAB in recurrent urinary tract infection *E. coli* strains. The investigation discovered that the most often observed TA system genes were relBE (11.3%), hipA (70.4%), mqsRA (43.1%), and ccdB (40.9%), which is inconsistent with our findings [26]. 

A study conducted in Mexico in 2023 discovered that all the uropathogenic *E. coli* strains were able to form biofilms. Significant differences were xxfound among higher OD and antibiotic resistance to cefazolin (p=0.0395), ceftazidime (p=0.0302), and cefepime (p=0.0420) [27]. In 2025, an Iranian study examined and compared the characteristics of enteropathogenic *E. coli* isolates from patients who suffer from diarrhea versus isolates from patients without diarrhea. The study found that approximately 61% of the isolates formed moderate biofilms, while weak biofilm producers accounted for 27% [28]. 

The current study found a strong correlation (P<0.05) between TA-positive isolates and biofilm development, as biofilm formation is more common in TA-positive isolates than in TA-negative isolates. Tsilibaris et al. [10] found more data supporting the function of TA systems in biofilm development. They employed strain of *E.coli* called Δ5 which lacks the most-studied TA pairs: mazF/mazE, relE/relB, yoeB/yefM, yafQ/dinJ, and chpB. The researchers found that the five deletions did not affect the stress response of bacterial cells. However, based on their microarray results, Ren et al. [16] reported that the TA systems were important for bio?lm formation. The study found that removing these five TA systems reduced biofilm development after 8 hours, but enhanced it after 24 hours in Luria/Miller medium at 37°C. Their findings support the importance of TA pairs in biofilm development [29]. Kolodkin-Gal et al. [1] sought to determine which of the TA systems has a stronger role in biofilm production. Using* E. coli *deletion mutants, they observed a significant decrease in biofilm formation in both the DmazEF and the DdinJ-yafQ mutants early on at 8 hours and after 24 hours, compared to their parental strain. However, they discovered only a partial decrease in biofilm formation in the DyefM-yoeB, DchpBIK, and DrelBE mutants. Studies [1], [16] have found that if TA systems are abundant and functioning in clinical isolates of the bacteria, they might be employed as a novel antibacterial method: a chemical compound capable of neutralizing the impact of the antitoxin. Although resistance development cannot be ruled out, targeting TA systems may decrease the probability of resistance emergence compared with traditional antibiotics. and liberating the toxin which can kill the cell.

## Notes

### Authors’ ORCIDs

- Yousefi Avarvand A: https://orcid.org/0000-0002-3987-9820
- Ekrami A: https://orcid.org/0000-0001-6138-8203
- Akrami S: https://orcid.org/0000-0001-6643-140X

### Ethical approval

The study was approved by the Ethics Committee of the Ahvaz Jundishapur University of Medical Sciences, Ahvaz, Iran (ethics code: IR.AJUMS.REC.1401.193) according to the Declaration of Helsinki. In this study, no consent form was obtained from the patients.

### Funding

The Infectious and Tropical Diseases Research Center, Health Research Institute, Ahvaz Jundishapur University of Medical Sciences, Ahvaz, Iran, financially supported this project and was not involved in the investigation (OG-0114).

### Competing interests

The authors declare that they have no competing interests.

## Figures and Tables

**Table 1:**
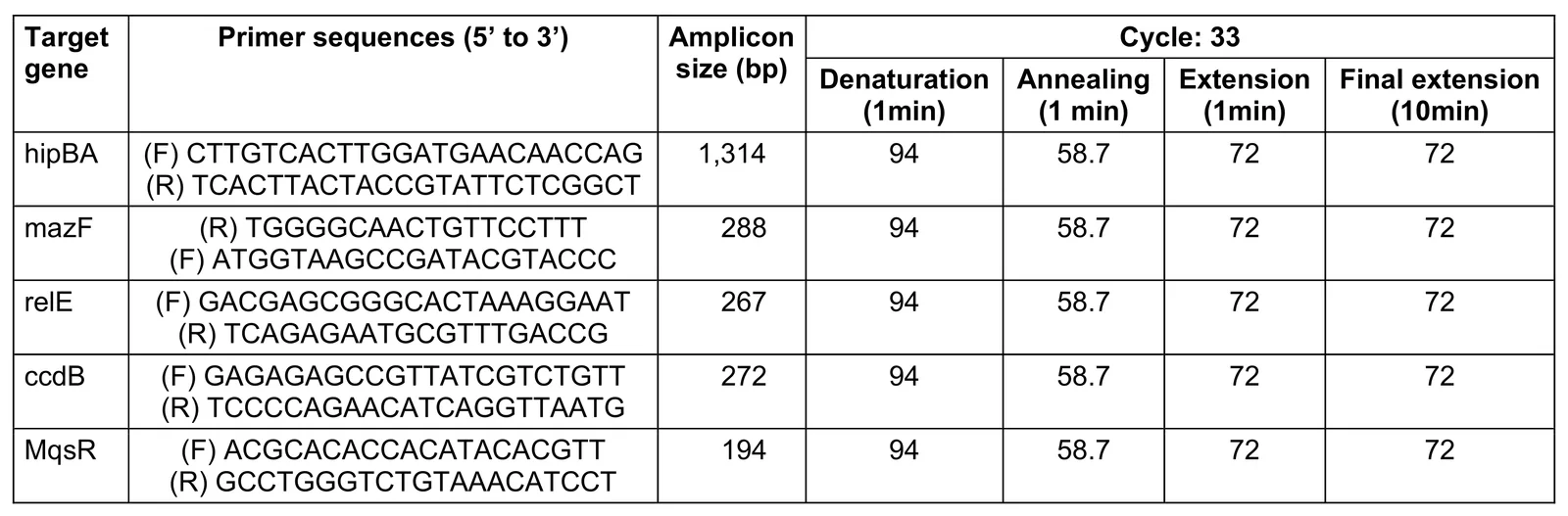
Primers used for detection of target genes and PCR programs for amplification
